# Effects of Transglutaminase Concentration and Drying Method on Encapsulation of *Lactobacillus plantarum* in Gelatin-Based Hydrogel

**DOI:** 10.3390/molecules28248070

**Published:** 2023-12-13

**Authors:** Junliang Chen, Zhiqin Liu, Shuhua Ma, Xin Chen, Linlin Li, Wenchao Liu, Guangyue Ren, Xu Duan, Weiwei Cao, Yunfeng Xu, Qinggang Xie

**Affiliations:** 1 College of Food and Bioengineering, Henan University of Science and Technology, Luoyang 471023, China; junliangchen@126.com (J.C.); linlinli2020@126.com (L.L.); wen_chaoliu@163.com (W.L.); guangyueyao@163.com (G.R.); duanxu_dx@163.com (X.D.); caoweiwei@haust.edu.cn (W.C.); 2 Heilongjiang Feihe Dairy Co., Ltd., Beijing 100015, China

**Keywords:** hydrogel encapsulation system, *Lactobacillus plantarum*, sodium hexametaphosphate, gelatin, transglutaminase, drying

## Abstract

*Lactobacillus plantarum* is a kind of probiotic that benefits the host by regulating the gut microbiota, but it is easily damaged when passing through the gastrointestinal tract, hindering its ability to reach the destination and reducing its utilization value. Encapsulation is a promising strategy for solving this problem. In this study, transglutaminase (TGase)-crosslinked gelatin (GE)/sodium hexametaphosphate (SHMP) hydrogels were used to encapsulate *L. plantarum*. The effects of TGase concentration and drying method on the physiochemical properties of the hydrogels were determined. The results showed that at a TGase concentration of 9 U/gGE, the hardness, chewiness, energy storage modulus, and apparent viscosity of the hydrogel encapsulation system were maximized. This concentration produced more high-energy isopeptide bonds, strengthening the interactions between molecules, forming a more stable three-dimensional network structure. The survival rate under the simulated gastrointestinal conditions and storage stability of *L. plantarum* were improved at this concentration. The thermal stability of the encapsulation system dried via microwave vacuum freeze drying (MFD) was slightly higher than that when dried via freeze drying (FD). The gel structure was more stable, and the activity of *L. plantarum* decreased more slowly during the storage period when dried using MFD. This research provides a theoretical basis for the development of encapsulation technology of probiotics.

## 1. Introduction

*Lactobacillus plantarum* can balance the intestinal microflora, improve immune function, and has antiobesity and antidiabetic properties [1]. However, most probiotics, including *L. plantarum*, encounter environmental stresses, such as high temperature, salt, oxygen, gastric acid, and bile salts, during food manufacturing, transportation, storage, and digestion in the human gastrointestinal tract, reducing their bioavailability and limiting their functional properties [2,3]. Microencapsulation technology can promote the long-term survival and the multiplication of bacteria in the human intestine. This technology is absorbent enough to release bacterial metabolites and fine solids, allowing probiotics to adhere to the colonic mucosa for long periods to colonize and grow [4]. But when probiotics are stored in the wet capsule state, probiotic activity tends to diminish due to environmental factors during transport, storage, and application. In contrast, the powder form is beneficial for maintaining their activity [5]. Therefore, further drying of microcapsules is required to improve performance indicators such as the survival of probiotics during long-term storage. The current drying techniques commonly applied to probiotics are spray drying (SD), vacuum freeze drying (FD), and microwave vacuum freeze drying (MFD) [5,6]. Although there are more studies on SD, the high temperature of SD harms the survival rate of probiotics. By comparison, FD needs a longer drying time and consumes more energy. Rodrigues et al. [7] used oligofructose and gum arabic as materials to encapsulate probiotics and fed them to 18 adult cats for 20 days. The results showed that the probiotic activity could be better maintained with FD than with SD. Ambros et al. [8] used MFD for *L. paracasei* and *Bifidobacterium* Animals. The results showed that the survival rate and membrane integrity after MFD were comparable to those of the conventional FD process, with survival almost maintained under all MFD process conditions for *Lactobacillus* and a 96% survival rate for *Bifidobacterium* under suitable process conditions.

Probiotic encapsulation technology was developed from the immobilized cell culture technology used in the biotechnology industry. The hydrogel protects probiotics from the harsh conditions of food processing [9,10,11]. At the same time, the gel matrix resists the chemical and enzymatic degradation of the carrier in the gastrointestinal tract. The hydrogel effectively releases its loaded biocomponents in the colon. It is one of the best probiotic protection mechanisms identified in the current literature [12]. The materials commonly used to prepare hydrogels are biocompatible and biodegradable macromolecules, such as animal and vegetable gums, proteins, and polysaccharides. GE-based hydrogels are inherently compatible with prominent molecules such as proteins and oligonucleotides [13]. The porous structure of GE-based hydrogels allows the loading and subsequent release of the active material. These hydrogels have been used as carriers of various bioactive compounds for the development of drug delivery systems [14]. Lu et al. [15] crosslinked yeasts with GE to improve the UV-blocking ability of hydrogel sunscreens. The sun protection factor could be increased from 0.75 to 29.42, and the cell survival rate was increased from 46.6% to 99% when the yeast concentration was increased from 0 to 2 g/mL. The release of the active substance could be adjusted by adjusting the degree of crosslinking, swelling, and degradation rate. Castro-Munoz et al. [16] applied a GE–maltodextrin composite as the encapsulation support to produce microcapsules of clarified juice from purple cactus pear. Ghosh et al. [17] investigated the chemical and drug release properties of thiamine hydrochloride and β-cyclodextrin host–guest solid inclusion compounds using a chitosan–GE hybrid network. Bini et al. [18] added curcumin and sodium naproxen to a GE matrix for two-drug encapsulation.

Recently, the complex coacervation method has mainly been used to prepare microcapsules by compounding GE with various anionic polymers, such as gum arabic, pectin, chitosan, extracellular polysaccharide, and alginate [19,20,21]. The dominant force driving the complex coacervation reaction relies on electrostatic interactions. Noncovalent interactions, such as hydrophobic interaction and hydrogen bonding, also contribute to the formation of coacervates [22]. Electrostatic interactions are usually weakened during thermal processing, which may eventually lead to the phase separation of the two biopolymers. Research showed that sodium hexametaphosphate (SHMP) is nontoxic and easy to gelate. SHMP can be hydrolyzed into simple phosphate in solution, which usually exists in the cell membrane of any organism. Therefore, SHMP is often used as a protein-modified material [23]. For instance, McCarthy et al. [24] reported that SHMP enhanced the functional properties of whey protein concentrate and reduced the dispersion viscosity. Rasouli et al. [25] found that the addition of SHMP could improve the protein stability and apparent viscosity. Furthermore, transglutaminase (TGase) can catalyze the acyl transfer reaction between the γ-hydroxylamine group of the glutamine residue in GE and the primary amine compound (acyl acceptor), resulting in covalent crosslinking and the ability to form strong gels [26].

The purpose of this study was to explore the development of conditions for obtaining a TGase-crosslinked GE/SHMP hydrogel with better characteristics. The textural properties, rheological properties, Fourier transform infrared spectra, fluorescence spectra, scanning electron microscopy, survival under simulated gastrointestinal conditions, and storage stability of the encapsulation systems were investigated. At the same time, FD and MFD techniques were used to prepare the encapsulation system. The drying mechanism and effects were compared and analyzed to provide theoretical support to further enrich and improve the drying technology used for probiotic microcapsules.

## 2. Results and Discussion

### 2.1. Textural Characterization

Textural properties are the most direct indicators used to evaluate the sensory performance of food. As can be seen from Table 1, the hardness, adhesiveness, and chewiness properties of the hydrogel encapsulation system increased first and then decreased as the concentration of TGase increased. The maximum hardness and chewiness were achieved at a TGase concentration of 9 U/gGE. When the concentration of TGase was higher than 9 U/gGE, excessive intramolecular covalent crosslinking was formed, which affected the orderly rearrangement of the GE network, preventing GE from forming a clear three-dimensional structure and causing reductions in hardness, adhesiveness, and chewiness. Chen et al. [27] used TGase and carrageenan to modify fish gelatin hydrogel and obtained similar results. The largest gel strength was achieved in the g-TG20 hydrogel, but it decreased with further increases in TGase concentration.

### 2.2. Dynamic Rheological Characterization

**Figure 1:**
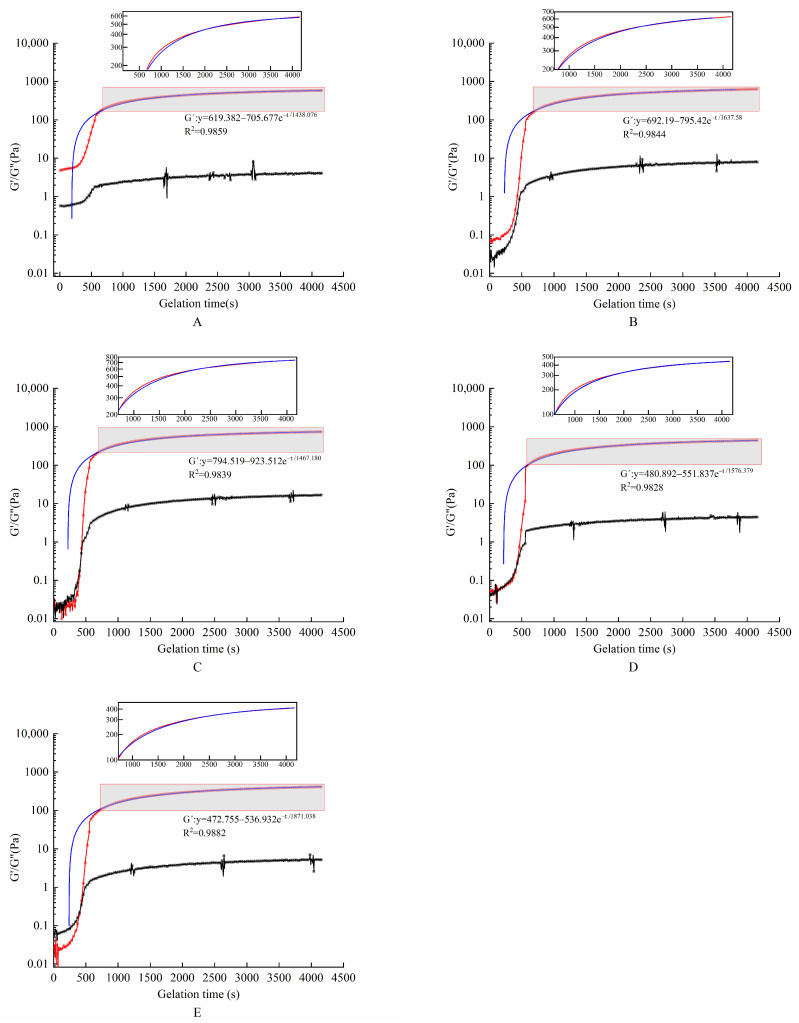
Evolution of storage and loss modulus during cooling and gelling of hydrogel encapsulation system with different enzyme concentrations. The red line represents the modulus of elasticity, the blue line represents the best linear fit for the modulus of elasticity, and the black line represents the modulus of loss. (**A**–**E**) Enzyme concentrations of 3, 6, 9, 12, and 15 U/gGE, respectively.

The effect of TGase concentration on the energy storage modulus G′ and the loss modulus G″ is shown in Figure 1. G′ and G″ for all gel systems grew at a fast rate during the first 10 min. G″ became higher than G′ after they intersected at a certain point, which indicated the formation of gel colloids during cooling. An analysis of Figure 1 and Table 2 shows that the G′ values of this hydrogel encapsulation system increased for all gel samples with different enzyme concentrations tested at dynamic scan times. This suggests that the interconnection of this envelope gradually formed over time in the presence of TGase. With the increase in TGase concentration, the G′ value and the rate of gel formation of the hydrogel encapsulation system increased first and then decreased. They reached a maximum when TGase was added at 9 U/gGE. As the enzyme concentration increased, the encapsulation system underwent excessive crosslinking. There was not enough time for the GE molecules to align with each other, leading to reductions in the G′ value and the rate of gel formation of hydrogel encapsulation system by affecting the connectivity of the gel network structure. Yan et al. [28] prepared an interpenetrating polymer network hydrogel using soy protein isolate (SPI) and sugar beet pectin (SBP) in combination with laccase to encapsulate *L. paracasei*. The results showed that the rheological properties were adjusted by adjusting the concentrations of SPI and SBP as well as the amount of laccase. The G′ value of the hydrogel increased with the increase in laccase concentration at first. When the laccase concentration reached 14 U/g, it rapidly crosslinked SPI and SPB. These two biopolymer molecules did not have enough time to align, resulting in lower G′ values.

### 2.3. Apparent Viscosity Analysis

**Figure 2:**
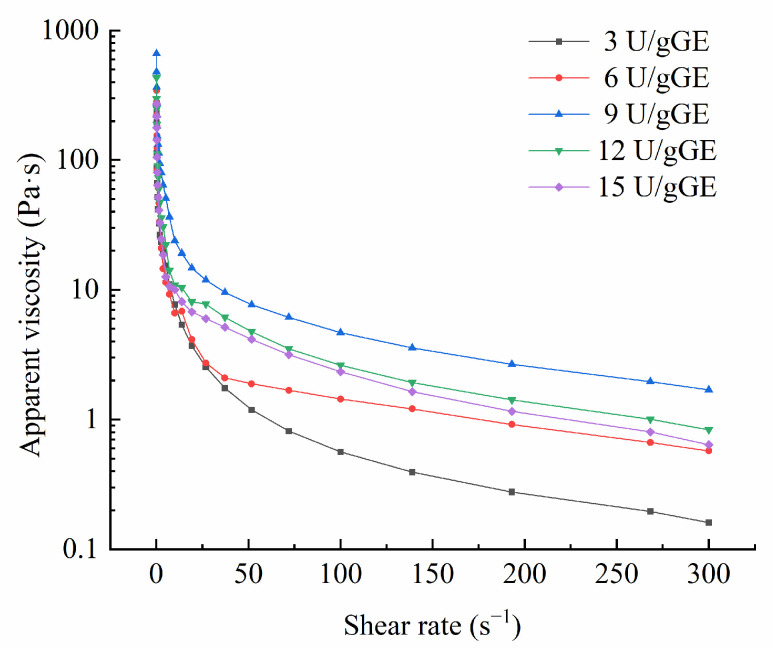
Effect of TGase concentration on apparent viscosity of GE/SHMP hydrogel encapsulation system.

In general, apparent viscosity can indicate changes in intermolecular forces. The more substantial the molecular attraction, the higher the apparent viscosity. Figure 2 presents the change in apparent viscosity of the hydrogel encapsulation system for different TGase concentrations. The graphs show that the apparent viscosity decreased with the increase in the shear rate, indicating that the hydrogel encapsulation system was a non-Newtonian pseudoplastic fluid. In addition, the apparent viscosity of this hydrogel was the highest at a TGase concentration of 9 U/gGE, which was conducive to catalyzing inter- and intramolecular crosslinking of this hydrogel to produce aggregates of high molecular weight.

The power-law, Bingham, and Herschel–Bulkley models were used to analyze the effect of different enzyme additions on the fluid properties of this hydrogel encapsulation system, and the power-law model better explained the rheological parameters than the Herschel–Bulkley, and Bingham models (R^2^ > 0.99). As can be seen from Table 3, the oscillation frequency of 50 s^−1^ and the c-value of the hydrogel encapsulation system increased and then decreased with increasing enzyme addition, with the maximum η50 and c-values found for 9 U/g GE, which could be attributed to the large number of intermolecular conjugations in the composite and the formation of large molecules catalyzed by TGase at higher concentrations in the hydrogel encapsulation system. Marcotte et al. [29] reported that when the fluid index (*p*) < 1, the fluid exhibited shear dilution and was a non-Newtonian fluid (pseudoplastic fluid). When *p* = 1, the sample was a Newtonian fluid; when *p* > 1, there was shear thickening, and the sample was an expansive plastic fluid. Power-law model analysis showed that the *p*-values for the hydrogel encapsulation system were all less than one, further confirming that it was a non-Newtonian fluid.

### 2.4. Secondary Structure Analysis

**Figure 3:**
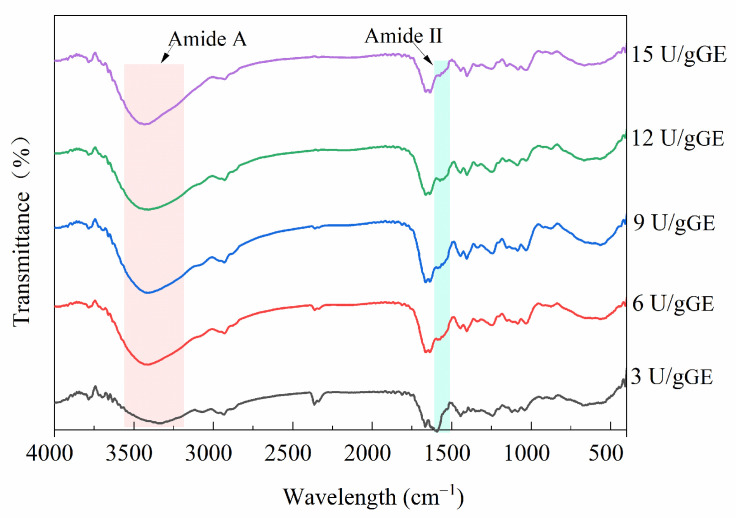
Effect of TGase concentration on secondary structural characteristics of GE/SHMP hydrogel encapsulation system.

Figure 3 shows the FTIR spectra of the effect of the TGase concentration on the structure of the hydrogel encapsulants. At an enzyme concentration of 3 U/gGE, this hydrogel encapsulant had a prominent peak at 1600 cm^−1^ to 1500 cm^−1^ (amide II), but it disappeared as the enzyme concentration increased. This was because when the degree of crosslinking was high, after TGase targeted the γ-COOH of glutamine and the ε-NH_2_ of lysine, the free amino group on the amide molecule was reduced. This result is in accordance with the result reported by Ahammed et al. [30], who showed that the peak amplitude of a TGase-modified film was lower than that of the control. Additionally, the characteristic peak of GE amide A usually appeared in the wavelength range of 3500–3100 cm^−1^. Figure 3 shows that the spectrograms of the hydrogels treated with different TGase concentrations also changed in the range of 3500–3100 cm^−1^. The shift in peak position with increasing enzyme concentration could be related to higher numbers of inter- and intramolecular hydrogen bonds. It could also be due to the exposure of aliphatic amino acid modifications and ε-(γ-glutamyl)-lysine crosslinking contributing to a more stable structure of the encapsulants. It was suggested that the formation of ε-(γ-Glu)-lys isopeptide bonds led to a reduction in hydrogen bonding and further led to a reduction in the triple-helix-like structure [31].

### 2.5. Tertiary Structural Analysis

**Figure 4:**
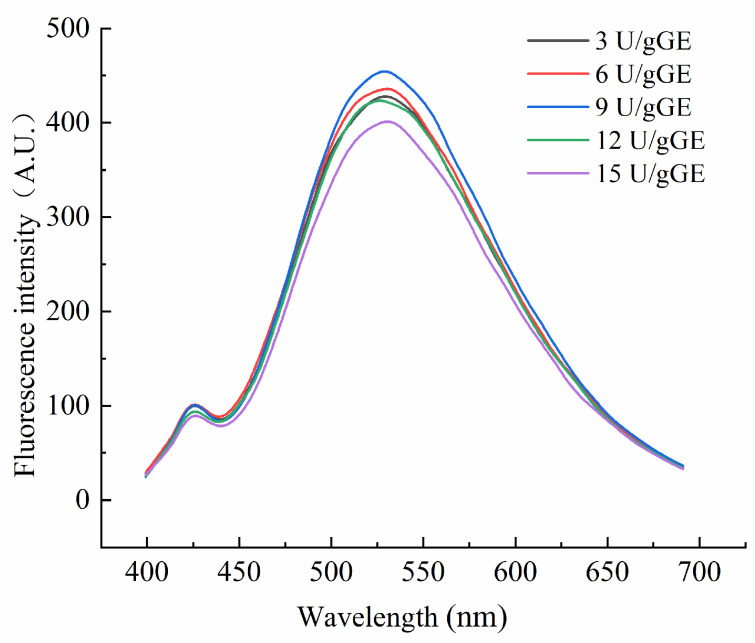
Effect of TGase concentration on tertiary structural characteristics of GE/SHMP hydrogel encapsulation system.

Endogenous fluorescence spectra reflect the effects of microscopic changes in external conditions on amino acids. Figure 4 shows the fluorescence spectra of the hydrogel encapsulants at different TGase concentrations. As can be seen, the maximum emission wavelength shifted blue as the enzyme concentration increased. It indicated a change in the polarity of the environment in which the amino acids were located. As the enzyme concentration increased, the crosslinking of the amino acids in the GE was more significant. But, at too high a concentration, the GE molecules became excessively entangled, and the maximum emission wavelength shifted red. TGase-mediated crosslinking at an enzyme concentration of 9 U/gGE enhanced the fluorescence intensity of the encapsulants, suggesting that the TGase crosslinking reaction at this concentration was more favorable for the exposure of specific fluorescent amino acid residues. This may be because, at an enzyme concentration of 9 U/gGE, the active group affected by TGase was exposed to more tryptophan. However, with the increase in enzyme concentration, the fluorescence intensity of the TGase crosslinked encapsulation system did not increase significantly but tended to weaken. This suggested that the unfolding of the protein–peptide chain during phosphorylation modification exposed more hydrophobic regions, and the hydrophobic groups formed aggregates under hydrophobic interaction forces. The hydrophobic groups were wrapped inside the molecule, resulting in a decrease in the fluorescence intensity.

### 2.6. Microstructural Analysis

**Figure 5:**
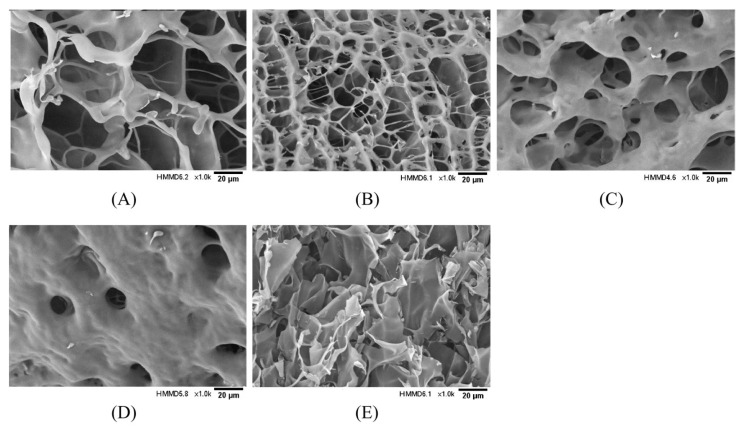
Effect of TGase concentration on microstructure of GE/SHMP hydrogel encapsulation system (×1.0 k). (**A**–**E**) Enzyme concentrations of 3, 6, 9, 12, and 15 U/gGE, respectively.

The effect of TGase concentration on the three-dimensional network structure of the hydrogel encapsulation system was observed using scanning electron microscopy, and the results are shown in Figure 5. With the increase in enzyme concentration, the hydrogel encapsulation system gradually changed from an irregular, large pore, relatively loose gel network structure to a honeycomb-like three-dimensional network structure with a smaller pore size and dense structure. Nevertheless, when the enzyme concentration was too high, the complex underwent excessive crosslinking, and the macromolecules became entangled. The network structure was disrupted, turning into irregular lamellar aggregates.

### 2.7. In Vitro Simulation of Gastrointestinal Bacterial Viability Analysis

In the simulated gastrointestinal tract, the effect of TGase concentration on the viability of *L. plantarum* in the hydrogel was as shown in Table 4. At the start of digestion, the live counts of *L. plantarum* ranged from 8.657 to 8.801 log CFU/mL. After digestion in the simulated gastric juice, the number of viable *L. plantarum* surviving in the encapsulated system ranged from 75% to 95%, and the number of viable bacteria in the simulated gastric juice tended to increase first and then decrease as the enzyme concentration increased; the lower the enzyme concentration, the faster the bacterial activity decreased. It was found that the release of *L. plantarum* in simulated gastric juice could be minimized at higher concentrations of TGase.

After 1 h of digestion in simulated intestinal fluid, the viable counts of *L. plantarum* decreased in all types of hydrogels but at a lower rate than in the simulated gastric fluid, suggesting that the *L. plantarum* in this hydrogel was more sensitive to the simulated gastric fluid. Additionally, the hydrogel encapsulation system was digested in simulated intestinal fluid for 2 h with only minimal reduction in *L. plantarum* bacterial activity at an enzyme concentration of 9 U/gGE. This indicated that the hydrogel provided good protection for *L. plantarum* at this concentration and was effective in transporting it to the large intestine.

### 2.8. Effect of TGase Concentration on the Storage Stability of L. plantarum

**Figure 6:**
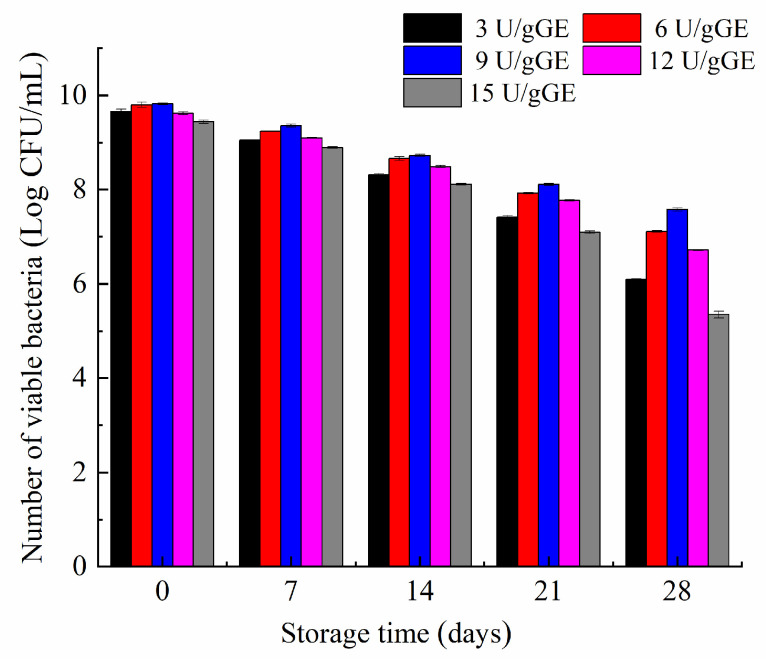
Effect of TGase concentration on storage stability of *Lactobacillus plantarum*.

The stability of *L. plantarum* in the hydrogel encapsulation system was determined periodically for 28 days. Figure 6 shows that the hydrogel with an enzyme concentration of 9 U/gGE only decreased viability by 2 log during storage. In contrast, the number of *L. plantarum* decreased more significantly in the hydrogel with lower or higher enzyme concentrations. This may have been because the hydrogel had a honeycomb structure and dense pore wall thickness at the concentration of 9 U/gGE, effectively reducing the loss of *L. plantarum* activity caused by hydrogel dehydration, thus showing the best water-holding capacity. It further showed that the gel with a dense network structure had a better protective effect.

### 2.9. Effect of Drying Method on the Crystal Structure of the Encapsulation System

**Figure 7:**
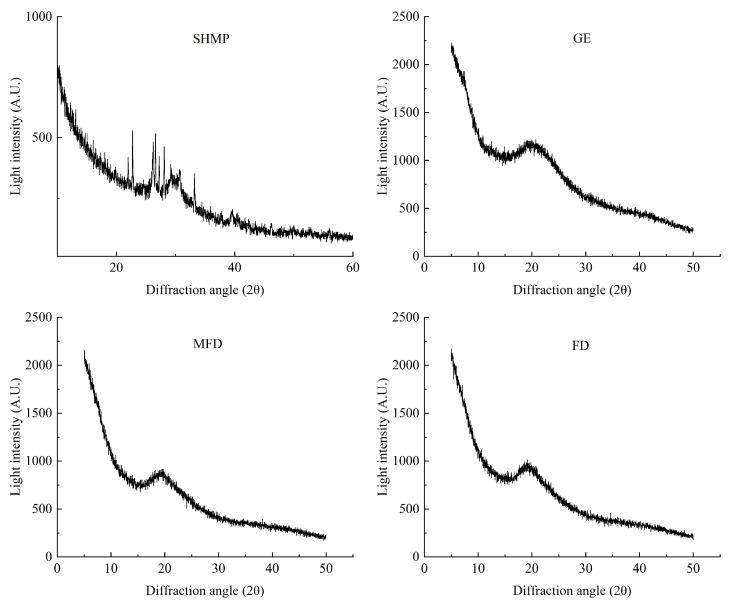
X-ray diffraction pattern of encapsulation system under drying.

X-ray diffraction (XRD) is one of the standard techniques used to verify the crystalline–amorphous state of dry components. The differences in the triple helix structure in samples were characterized using XRD, and the results are shown in Figure 7. A distinct diffraction peak was observed in the X-ray diffractogram of GE at about 20° (2θ), which is similar to previously reported results [32]. SHMP had many sharper diffraction peaks, which were formed by different phosphate groups. Additionally, it can be seen that the intensity of the diffraction peaks of the encapsulation system was lower after drying, and the polymer formed a semicrystalline structure with a slightly sharp diffraction peak. This may have been because TGase promoted the spatial site block of ε-(γ-Glu)-lys isopeptide bonds and limited the formation of hydrogen bond in the gel, leading to a decrease in the number of triple-helix-like structures [33]. However, there was a minor impact on crystal type and crystallinity of the encapsulation system dried via MFD and FD.

### 2.10. Effect of Drying Method on the Thermal Stability of the Encapsulation System

**Figure 8:**
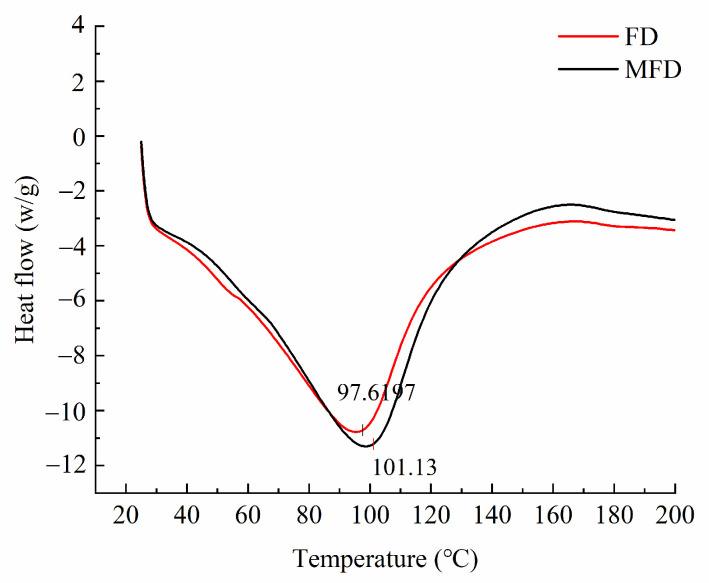
Thermal stability of encapsulation system under different drying methods.

In order to conduct an in-depth evaluation of the differences in the thermal stability of the encapsulation system after drying using different methods, differential scanning calorimetry (DSC) analysis was performed. As shown in Figure 8, the thermal denaturation temperature of the FD encapsulation system was slightly lower than that of the MFD encapsulation system, indicating that microwave as an auxiliary heat source shortened the drying cycle and reduced the exposure time of the hydrogel encapsulation system under the harsh environment, thus reducing its denaturation to some extent.

### 2.11. Effect of Drying Method on the Moisture Distribution Status and Storage Stability of the Encapsulation System

**Figure 9:**
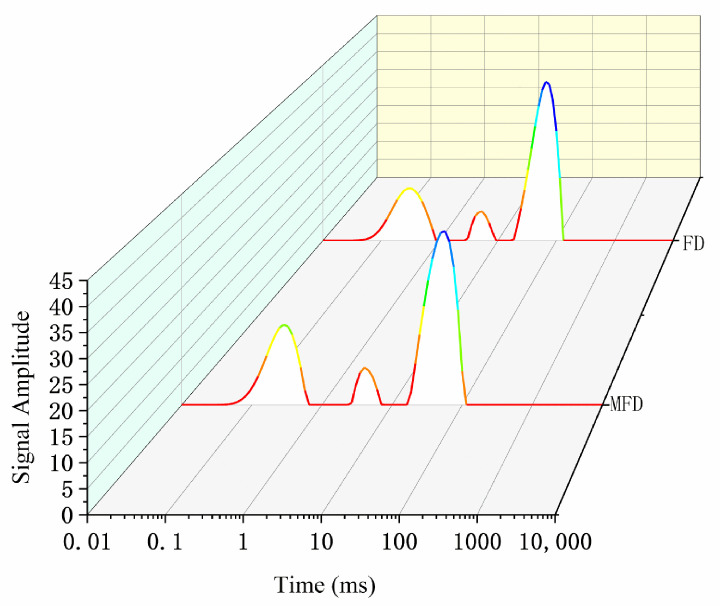
Moisture distribution status under different drying methods.

Low-field nuclear magnetic resonance (NMR) can reflect the mobility and ratio of various water molecule fractions within an encapsulation system without destroying the gel structure. The relaxation time (T_2_) contains three components: bound water (T_21_), immobilized water (T_22_), and free water (T_23_) [34]. Usually, the relaxation time of a gel is inversely proportional to the binding degree of the water molecules due to the co-oscillation of water molecules [35]. The higher the degree of freedom, the higher the co-oscillation frequency compared with that of hydrogen protons, resulting in an increase in relaxation time. As shown in Figure 9 and Table 5, the encapsulation system dried via MFD had a shorter relaxation time than that dried via FD, which indicated that the degree of freedom of water was lower after drying using the MFD method. Additionally, the MFD sample had a higher ratio of bound water (30.82%) and lower ratio of free water (61.86%) than the FD sample. This suggested that the system, after MFD, was more favorable for the long-term stabilization of *L. plantarum* compared with that after FD.

Table 6 shows that the viability of *L. plantarum* dried using MFD decreased less than that dried using FD during storage. This is because FD was performed at low temperatures for a long time, and mechanical stress occurred during the formation of ice crystals inside the cells. Mechanical stress and high water content could have decreased the viability of *L. plantarum* during long-term storage. Furthermore, the viability of *L. plantarum* was not only dependent on temperature but also on water activity and moisture content during processing and subsequent storage as well. Ananta and Teixeira stated that to maintain stability after drying, the water activity should not exceed 0.25, and the moisture content should be 4–7%, as appropriate [36,37].

## 3. Materials and Methods

### 3.1. Materials

MRS broth and solid medium were purchased from Beijing Aubergine Biotechnology Co. (Beijing, China). SHMP was purchased from Tianjin Deyen Chemical Reagent Co. (Tianjin, China). GE (Power 160) was bought from Zhengzhou Tianshun Food Additives Co. (Zhengzhou, China). TGase (120–138 U/g) was obtained from Jiangsu Yiming Biological Co. (Taizhou, China). *L. plantarum* was isolated from Chinese sauerkraut and conserved by our laboratory. Pepsin (1:30,000), trypsin (BR, 1:250), and bile salts (≥60% bile acid) were bought from Shanghai Yuanye Biotechnology Co. (Shanghai, China). Disodium hydrogen phosphate, sodium dihydrogen phosphate, glacial acetic acid, and hydrochloric acid were of analytical grade.

### 3.2. Preparation of Bacterial Suspension

*L. plantarum* stored at −80 °C was inoculated in MRS broth medium at 2% (*v*/*v*), incubated for 2–3 generations, and then collected from the prestationary stage, centrifuged for 10 min (4 °C, 5000 r/min), and resuspended in sterile NaCl (0.75%, *w*/*v*) solution. The number of bacteria in the suspension was around 10^8^–10^9^ CFU/mL. The suspension was stored at 4 °C until use.

### 3.3. Preparation of Composite Hydrogel Encapsulants

Phase I: For the preparation of phosphorylated GE, we referred to the method of Cen et al. [38]. Briefly, a 3.5% (*w*/*v*) GE solution was dissolved by stirring at a certain speed for 20 min at 40 °C in a water bath. Then, SHMP was added at a GE/SHMP ratio of 35/1 and stirring was continued to mix them well, at which point the solution formed a stable emulsion. A 10% (*v*/*v*) acetic acid solution was added dropwise to the emulsion under continuous stirring. The pH of the solution was adjusted to 4.8 to crosslink the GE and SHMP to form a primary hydrogel, which was left overnight in a refrigerator at 4 °C after 30 min of reaction.

Phase II: For the preparation of the hydrogel encapsulation system, we referred to the method of Yan et al. [28]. The above prepared hydrogel was stirred at a certain speed and added to the bacterial suspension at a ratio of 1:4 (hydrogel/bacterial suspension, *v*/*v*) at 40 °C. At the same time, 100 μL of Tween-80 was added as emulsifier and stirred for 10 min so that the bacterial suspension was fully adsorbed in the gel network. Then, different concentrations of TGase were added, and the wet microcapsules were collected for analysis after stirring for 2 h at 40 °C in a water bath.

### 3.4. Drying of Composite Hydrogel Encapsulation Systems

#### 3.4.1. Vacuum Freeze Drying

The wet-based hydrogel encapsulation systems prepared above were laid in a 20 mm Petri dish with a thickness of about 2 cm and dried using a vacuum freeze dryer. The wet microcapsule samples were prefrozen in a freezer for about 6 h, after which they were dried in a drying chamber under 40 Pa pressure with the cold trap and hot rack temperatures set to −40 °C and −50 °C, respectively. The FD (LGJ-10D, Beijing Scientific Instruments Co., Ltd. Beijing, China) microcapsule samples were stored in a desiccator for analysis.

#### 3.4.2. Microwave Vacuum Freeze Drying

The above-prepared wet-based hydrogel encapsulation systems were laid in a Petri dish in a thickness of 2 cm. They were put into a refrigerator at −18 °C for 12 h for prefreezing. Before the encapsulation systems were put into the MFD (laboratory homemade) dryer, the temperature of the cold refrigerator was dropped to −40 °C. The vacuum pump was turned on, and the vacuum level was set at 100 Pa. The microwave system was turned on, and the microwave power was set at 0.6 W/g until the moisture content of the materials was less than 0.13 g/g (on a wet basis)

### 3.5. Determination of Textural Properties

The gel samples with TGase concentrations of 3, 6, 9, 12, and 15 U/gGE were poured into gel molds and cut into cylinders 20 mm in height after 12 h. A P/0.5 cylindrical probe (TA. XT Express, Stable Micro Systems, Godalming, UK) was used with a pretest rate of 2.00 mm/s, a test rate of 1.00 mm/s, a return rate of 1.00 mm/s, a trigger force of 5 g, and a compression deformation degree of 40%. Each set of samples was tested three times, and the average value was taken as the experimental result.

### 3.6. Measurement of Rheological Properties

#### 3.6.1. Dynamic Rheological Properties Test

The dynamic rheological properties were tested referring to the procedure of Hu et al., with some modifications [39]. Gel samples with TGase concentrations of 3, 6, 9, 12, and 15 U/gGE were scanned using a rheometer (DHR-2, Waters Co., Ltd. Milford, CT, USA) using a 40 mm diameter plate, set at a measurement spacing of 1 mm, a frequency of 1 Hz, and a linear viscoelastic range of 1.0%. The GE solution was cooled from 50 °C to 4 °C, followed by a scanning temperature of 4 °C for 3600 s.

(1) $$G_{t}^{\prime }\;(\mathrm{Pa})=C+K_{\mathrm{gel}}e^{-t/A}$$

where K_gel_ is the gelation speed; t is the gelation time; A and C are constants.

#### 3.6.2. Shear Rate Test

The shear rate was tested with reference to the method of Hu et al. [39]. The fluid properties of the hydrogel encapsulation system were analyzed using the power-law, Bingham, and Herschel–Bulkley models. The analysis was carried out as follows:

Power-law model: τ (Pa) = c·γ*^p^* (2)

Bingham model: τ (Pa) = τ_B_ + η_B_·γ (3)

Herschel–Bulkley model: τ (Pa) = τ_HB_ + c·γ*^p^* (4)

where τ is the shear force; c is the flow coefficient; γ is the shear rate; *p* is the flow index; τ_B_ is the yield stress; η_B_ is the Bingham flow coefficient; and τ_HB_ is the yield stress of the Herschel–Bulkley model.

### 3.7. Measurement of Fourier Transform Infrared Spectra

The MFD samples were mixed and ground with KBr at a ratio of 1:100 after MFD. The FTIR spectra were measured using a spectrometer (VERTEX70, Brucker, Karlsruher, Germany). The scanning range was 4000 to 400 cm^−1^ with a resolution of 4 cm^−1^, for a total of 64 scans.

### 3.8. Determination of Fluorescence Spectra

The endogenous fluorescence spectra were measured with reference to the method of Chen et al. [40]. Fresh samples of the hydrogels were placed in quartz test tubes. The endogenous fluorescence in the hydrogels was measured using a fluorescence spectrophotometer (Cary eclipse, Aglient Technologies Co., Ltd. Santa Clara, CA, USA) at an excitation wavelength of 380 nm and an emission wavelength of 400–700 nm. The slit widths for both excitation and emission were 10 nm. Phosphate buffer (pH 7.0) was used as a control group and all samples were measured three times.

### 3.9. Determination of Microstructure

The samples were fixed to a holder with carbon conductive tape at accelerating voltages of 10 and 15 kV. They were plated with gold using a vacuum sputter-coater. The surface structure of the microwave vacuum freeze-dried encapsulation system was scanned using a scanning electron microscopy (TM3030Plus, Hitachi High-Technologies Co., Ltd. Tokyo, Japan) at 500× magnification.

### 3.10. Survival under Simulated Gastrointestinal Conditions

For the simulated gastrointestinal condition, we referred to the method of Su et al. [41]. The simulated gastric juice was prepared as follows: 0.85% sterile saline was adjusted to pH 2.0 with 1.0 mol/L HCl solution, and then pepsin at the concentration of 3 g/L was added. For the simulated intestinal solution, 0.85% sterile saline was adjusted to pH 6.8; then, 1.0 mol/L NaOH solution, 4.5 g/L bile salts, and 1 g/L pancreatic enzyme were added. A 30 mL sample of the gel was added to 30 mL of simulated gastric juice, and then the mixture was placed in a shaking incubator at 37 °C. Samples were taken after 60 min for determination. The digested mixture was adjusted to pH 6.8 with 10% sodium oxide and mixed with an equal amount of simulated intestinal fluid. The mixture was incubated for 2 h at 37 °C with shaking, and samples were taken in 60 min intervals for determination.

### 3.11. Determination of Crystal Structure

The powder samples dried using different methods were inserted into aluminum holders using glass sheets, and the samples were slightly compressed for XRD measurements (D8 ADVANCE, Brucker, Karlsruher, Germany).

### 3.12. Measurement of Thermal Stability

DSC was adopted to measure the thermal stability of the encapsulation system dried using different methods. The samples were weighed, and 10 mg from each group was pressed in an aluminum crucible. The thermal stability analysis was carried out by setting the starting temperature to 25 °C, the ending temperature to 200 °C, with a scanning temperature range of 20 to 200 °C, and the scanning rate to 10 °C/min to obtain the DSC (DSC1, Mettler-Toledo, Zurich, Switzerland) curve.

### 3.13. Determination of Moisture Distribution State

The prepared fresh samples were placed in NMR vials and stored in a refrigerator at 4 °C for 12 h. The samples were placed in the center of a radiofrequency coil and measured using the CPMG procedure. The measurement conditions were as follows: 4000.0 ms sampling time interval, 8 accumulations, 1 preamplification, and 10,000 echoes. After sampling, the CPMG index decay curve was inverted using Newmark NMR inversion software (Shanghai Electronic Technology, Co., Ltd., Shanghai, China) to obtain the low-field NMR relaxation time spectra.

### 3.14. Determination of Storage Stability

The gel samples were stored at 4 °C for 28 days. The survival rate of *L. plantarum* during storage was determined.

### 3.15. Statistical Analysis

Plots were created and analysis of variance (ANOVA, *p* < 0.05) was performed using OriginPro 2021 (Origin Lab Co., Ltd. Northampton, MA, USA) and SPSS Statistics 20 software (International Business Machines Co., Ltd. Armonk, NY, USA).

## 4. Conclusions

The effects of TGase concentration and drying method on the performance of GE/SHMP hydrogels were studied. It was found that the energy storage modulus and apparent viscosity of the encapsulation system were maximized at an enzyme concentration of 9 U/gGE, which was conducive to the intermolecular action and connection during the formation of the encapsulation system, resulting in the formation of a hydrogel encapsulation system with the greatest hardness, adhesiveness, and chewiness. The analysis of the secondary, tertiary, and micro structure of the encapsulation system further showed that the surface could form a more stable molecular structure at this concentration, and the gel network structure was dense, which was conducive to the encapsulation of *L. plantarum*. These findings were further confirmed through the determination of gastrointestinal digestive stability and storage stability of the hydrogel encapsulation system, which showed improved survival under simulated upper gastrointestinal conditions at an enzyme concentration of 9 U/gGE and higher viability of *L. plantarum* after 28 days of storage at 4 °C. The thermal stability of the encapsulation system dried via MFD was slightly higher than that of the system dried via FD with reduced water mobility and a slighter decrease in bacterial activity during storage. The results of this study therefore suggest that the TGase-crosslinked GE/SHMP hydrogel could serve as a delivery system for functional components and the controlled release of probiotics. The future development of this system involves exploring and analyzing the digestive properties and colonization potential of probiotic microcapsules in the gastrointestinal tract of mice, as well as its impact on intestinal microecology.

## Figures and Tables

**Table 1:** Effect of TGase addition on the constitution characteristics of composite hydrogel encapsulation system. Different letters indicate significant differences (*p* < 0.05).

TGase Concentration (U/gGE)	Hardness (g)	Adhesiveness (g.s)	Springiness (%)	Chewiness (N)	Resilience
3	677.835 ± 6.290 ^e^	−114.114 ± 0.288 ^d^	0.957 ± 0.006 ^c^	383.671 ± 1.228 ^e^	0.287 ± 0.011 ^c^
6	1408.065 ± 20.913 ^b^	−82.245 ± 7.297 ^c^	0.964 ± 0.002 ^c^	1145.251 ± 13.620 ^b^	0.577 ± 0.008 ^b^
9	2296.01 ± 45.415 ^a^	−10.492 ± 1.029 ^a^	0.997 ± 0.001 ^a^	1898.900 ± 1.878 ^a^	0.567 ± 0.007 ^b^
12	1084.324 ± 9.837 ^c^	−48.062 ± 6.584 ^b^	0.981 ± 0.015 ^b^	884.923 ± 5.865 ^c^	0.591 ± 0.011 ^b^
15	875.680 ± 1.780 ^d^	−18.837 ± 7.047 ^a^	0.987 ± 0.006 ^ab^	792.439 ± 4.724 ^d^	0.638 ± 0.018 ^a^

**Table 2:** Effect of TGase concentration on the gel kinetics of complex hydrogel encapsulation system.

TGase Concentration (U/gGE)	K_gel_ (Pa/s)	R^2^	G′_Final_ (Pa)
3	705.677	0.9859	588.033
6	795.420	0.9844	631.113
9	923.512	0.9839	774.231
12	551.837	0.9828	445.022
15	536.932	0.9882	413.111

**Table 3:** Analysis of power-law model fitting parameters.

Power-Law Model	TGase Concentration (U/gGE)				
	3	6	9	12	15
c	42.162	67.306	117.173	68.074	52.565
*p*	−0.763	−0.722	−0.728	−0.785	−0.720
R^2^	0.999	0.994	0.991	0.996	0.994
η50	1.18682	1.88556	7.69007	4.76029	4.15941

**Table 4:** Effect of TGase concentration on the viability of *Lactobacillus plantarum* in vitro to mimic the gastrointestinal tract. Different letters indicate significant differences (*p* < 0.05).

Time (min)	Viability of *L. plantarum* (Log CFU/mL)				
	3 U/gGE	6 U/gGE	9 U/gGE	12 U/gGE	15 U/gGE
0	8.657 ± 0.049 ^c^	8.801 ± 0.054 ^b^	8.697 ± 0.058 ^ab^	8.786 ± 0.041 ^b^	8.778 ± 0.050 ^ab^
60	6.521 ± 0.114 ^c^	8.095 ± 0.030 ^a^	8.237 ± 0.019 ^a^	8.103 ± 0.025 ^a^	7.864 ± 0.078 ^b^
120	6.052 ± 0.279 ^c^	7.949 ± 0.043 ^a^	8.129 ± 0.022 ^a^	7.880 ± 0.072 ^a^	7.367 ± 0.107 ^b^
180	5.537 ± 0.080 ^d^	7.091 ± 0.066 ^b^	7.4114 ± 0.014 ^a^	7.058 ± 0.035 ^b^	6.580 ± 0.057 ^c^

**Table 5:** Effect of drying method on the moisture distribution status of the encapsulation system.

Drying Method	Relaxation Time (ms)			Content (%)		
	T_21_	T_22_	T_23_	A_21_	A_22_	A_23_
MFD	0.645	5.801	98.678	30.82	7.32	61.86
FD	0.857	6.67	115.896	28.10	7.45	64.45

**Table 6:** Effect of drying method on storage stability of the encapsulation system.

Storage Time (Days)	Bacterial Activity Count (Log CFU/g)				
	0	7	14	21	28
FD	7.685 ± 0.036	7.908 ± 0.040	7.430 ± 0.025	7.058 ± 0.034	6.544 ± 0.060
MFD	7.599 ± 0.046	7.687 ± 0.045	7.455 ± 0.013	7.223 ± 0.017	6.827 ± 0.027

## Data Availability

The data used to support the results of this study can be provided by the corresponding authors upon request.
